# Knowledge-based expedited parameter tuning of microwave passives by means of design requirement management and variable-resolution EM simulations

**DOI:** 10.1038/s41598-023-27532-4

**Published:** 2023-01-06

**Authors:** Slawomir Koziel, Anna Pietrenko-Dabrowska, Ali Ghaffarlouy Raef

**Affiliations:** 1 Engineering Optimization and Modeling Center, Reykjavik University, 102 Reykjavik, Iceland; 2 Faculty of Electronics, Telecommunications and Informatics, Gdansk University of Technology, 80-233 Gdansk, Poland

**Keywords:** Electrical and electronic engineering, Computational science

## Abstract

The importance of numerical optimization techniques has been continually growing in the design of microwave components over the recent years. Although reasonable initial designs can be obtained using circuit theory tools, precise parameter tuning is still necessary to account for effects such as electromagnetic (EM) cross coupling or radiation losses. EM-driven design closure is most often realized using gradient-based procedures, which are generally reliable as long as the initial design is sufficiently close to the optimum one. Otherwise, the search process may end up in a local optimum that is of insufficient quality. Furthermore, simulation-based optimization incurs considerable computational expenses, which are often impractically high. This paper proposes a novel parameter tuning procedure, combining a recently reported design specification management scheme, and variable-resolution EM models. The former allows for iteration-based automated modification of the design goals to make them accessible in every step of the search process, thereby improving its immunity to poor starting points. The knowledge-based procedure for the adjustment of the simulation model fidelity is based on the convergence status of the algorithm and discrepancy between the current and the original performance specifications. Due to using lower-resolution EM simulations in early phase of the optimization run, considerable CPU savings can be achieved, which are up to 60 percent over the gradient-based search employing design specifications management and numerical derivatives. Meanwhile, as demonstrated using three microstrip circuits, the computational speedup is obtained without design quality degradation.

## Introduction

Geometries of microwave passive components become continuously more involved to fulfil the performance requirements of various application areas (wireless communications including 5G and 6G^1,2^, internet of things^3^, wireless sensing^4^, microwave imaging^5^, wearable devices^6^, autonomous vehicles^7^, etc.). In particular, many of these applications require specific functionalities such as multi-band operation^8^, harmonic suppression^9^, customized phase characteristics^10^, reconfigurability^11^, often combined with limitations on the physical size of the devices^12–15^. Due to their inherent complexity, microwave structures designed to meet these and other requirements typically feature considerably increased numbers of design variables than conventional circuits, whereas their design process has to account for several objectives and constraints imposed on their electrical characteristics. Adequate parameter tuning of such circuits requires rigorous numerical optimization^16–20^. At the same time, the adjustment process has to be carried out at the level of full-wave electromagnetic (EM) simulations to account for effects such as EM cross-couplings, or dielectric and radiation losses. These cannot be properly quantified using analytical or equivalent network models, yet are important for the operation of modern circuits implemented using techniques such as transmission line folding^21^, defected ground structures^22^, the employment of slow-wave phenomena (e.g., compact microstrip resonant cells, CMRCs^23^), or the incorporation of geometrical modifications (stubs^24^, slots^25^, shorting pins^26^). Although imperative from the standpoint of ensuring design quality, EM-driven design optimization is computationally expensive, even in the case of local tuning^27^, let alone global^28^ or multi-objective search^29^, or statistical design^30^.

Given the importance of simulation-based design, substantial research efforts have been aimed at addressing the underlying challenges, primarily in terms of improving the computational efficiency of the optimization procedures. In the case of local gradient-based search, the major bottleneck is the evaluation of the system response gradients, which can be accelerated using adjoint sensitivities^31,32^, by restricting the finite-differentiation-based updates^33–35^, or utilization of mesh deformation techniques^36^. In some cases, the cost of simulation-driven design may be reduced by the employment of fast dedicated solvers^37^. Another option is the exploration of the specific structure of the system response (e.g., the allocation of resonances, in-band ripples, etc.) using techniques such as response feature technology^38,39^, or cognition-driven design^40^. Notwithstanding, one of the most popular approaches in the recent years have become surrogate-assisted methods^20,41,42^. Therein, most of the operations are carried out at the level of fast surrogates, with costly EM simulations only executed occasionally, to validate the designs produced using the metamodels or to obtain the data necessary for model refinement. Among the two major classes of surrogate modelling methods, the physics-based ones are more often used for local search purposes (space mapping^43^, response correction^44–46^), whereas data-driven models (kriging^47^, Gaussian process regression, GPR^48^, artificial neural networks^49–51^, support vector regression^52^, polynomial chaos expansion^53^) are perceived as more generic, and suitable for global and multi-criterial design^54–56^, as well as uncertainty quantification^57–60^. Related methods include machine learning techniques^61–63^, as well as surrogate-assisted frameworks involving variable-resolution models (two-level GPR^64^, co-kriging^65^).

While the methods outlined in the previous paragraph mainly focus on reducing the CPU costs, reliability of simulation-based design procedures is just as important consideration. In practice, the lack of sufficiently good initial design may lead to a failure of local parameter tuning, with the alternative being the involvement of much more expensive global search algorithms. A typical situation is dimension scaling (re-design of a circuit to meet different operating parameters, e.g., the centre frequency or dielectric substrate), or design of miniaturized components employing CMRCs^66^. Although global search may be accelerated using surrogate-assisted methods^67,68^, these methods are incapable of handling structures featuring large number of parameters^69^.

An attempt to address the reliability issues pertinent to local search procedures has been made in a recently introduced design requirement management procedure^70^. Therein, the design goals for a given iteration of the optimization algorithm are set up having in mind the actual operating parameters of the circuit at hand. In particular, the goals (e.g., target operating frequencies) are relocated automatically to ensure their attainability through local tuning. As the optimization process progresses, the objectives step-by-step converge to their initial values. The method of Koziel et al.^70^ has been shown to significantly enhance the immunity of the gradient-based algorithms to inferior-quality starting points at the expense of a certain increase of the computational cost. In this paper, we describe a novel procedure, which is an advancement over^70^ in terms of improving the computational efficacy of the search process. The latter is achieved via the incorporation of variable-fidelity EM models, selected from a continuous spectrum of the assumed resolutions. A priori knowledge concerning the range of admissible model resolutions is necessary to set up the proposed optimization framework, and it has to be provided by the user. Typically, this range is assessed through grid convergence studies, i.e., visual inspection of the families of component responses at various fidelities. The lowest-fidelity simulations are utilized at the onset of the optimization process, which allows for parameter space exploration at minimum CPU expenses. During the algorithm course, the problem-specific knowledge (in the form of the actual operating parameters of the current design) is extracted from the EM-simulated components response at this design, and, similarly as in Koziel et al.^70^, is subsequently utilized to adjust the design goals at the current iteration, as well as the EM model fidelity. As the design goals (according to the scheme adopted from^70^) become closer to their original values, and the algorithm starts to converge—as measured by the relocation of the design and iteration-wise objective function differences—the model fidelity increases, to eventually attain the high-fidelity level near the conclusion of the run. The proposed knowledge-based procedure has been verified using three microstrip circuits, including two couplers and a dual-band power divider. The obtained results demonstrate a significant improvement of the computational efficiency with the average savings of 55 percent over the single-fidelity procedure of Koziel et al.^70^, and essentially no detrimental effects on the design quality. The presented algorithm offers improved reliability under difficult design scenarios (e.g., poor initial conditions), as well as reduced running costs. The former feature extends the applicability of local search procedures by reducing the need to default to global routines, which is of significant practical importance. The aforementioned advantages of the introduced procedure, i.e., its enhanced reliability and increased computational efficacy, have been achieved by employing two mechanisms, automated design requirement management and knowledge-based adjustment of the simulation model fidelity.

The novelty and the technical contribution of this work can be summarized as follows: (1) development and implementation of a variable-resolution design optimization framework algorithm with design requirement management, (2) association of the model fidelity management scheme with the discrepancy between the target and actual operating frequencies of the component under design, (3) verifying reliability and low computational cost of the introduced algorithm under demanding scenarios, especially inferior-quality starting points operating at frequencies severely misaligned with the targets. The presented algorithm combines the computational efficiency and robustness, which are integrated in a single optimization procedure. To the authors’ best knowledge, no comparable algorithm for design optimization of microwave components has been reported in the literature thus far.

## Adaptive design requirements for reliability improvement

This section briefly summarizes the adaptive performance specification method of Koziel et al.^70^, which is one of the two major ingredients of the optimization framework proposed in the paper. The other, variable-fidelity model management, will be elaborated on in “Variable-fidelity models for optimization cost reduction” section.

### Automated adaptation of design requirements for local search enhancement

The design requirement management scheme of Koziel et al.^70^ will be explained using a design problem in which the *N*-band circuit at hand is to work at the target frequencies *f*_*k*_, *k* = 1. We denote by ***x*** a vector of design parameters, and by ***S***(***x***) the EM-evaluated outputs (normally, the scattering parameters). The frequencies *f*_*k*_ are gathered into a target vector ***F*** = [*f*_1_ … *f*_*N*_]^*T*^. The aim is to find

1 $${\boldsymbol{x}}^{*} = \arg \mathop {\min }\limits_{{\boldsymbol{x}}} U({\boldsymbol{S}}({\boldsymbol{x}}),{\boldsymbol{F}})$$

with *U* being a merit function (or objective function). For additional clarification, consider an equal-split coupler, which is to run at the operating frequency *f*_0_; the device is supposed to minimize both input matching and port isolation at *f*_0_. Given the above, the characteristics of interest are S-parameters ***S***_*j*1_, *j* = 1, …, 4. The function *U* can take the form of

2 $$\begin{gathered} U({\boldsymbol{S}}({\boldsymbol{x}}),{\boldsymbol{F}}) = U\left( {[S_{11} ({\boldsymbol{x}},f),S_{21} ({\boldsymbol{x}},f),S_{41} ({\boldsymbol{x}},f),S_{41} ({\boldsymbol{x}},f)],[f_{0} ]} \right) = \\ \max \left\{ {|S_{11} ({\boldsymbol{x}},f_{0} )|,|S_{41} ({\boldsymbol{x}},f_{0} )|} \right\} + \beta \left[ {|S_{21} ({\boldsymbol{x}},f_{0} )| - |S_{31} ({\boldsymbol{x}},f_{0} )|} \right]^{2} \\ \end{gathered}$$

Note that the objectives are categorized: minimization of |*S*_11_| and |*S*_41_| is the primary goal; the equal power split condition is an equality constraint, ensured by the penalty term proportional to the penalty coefficient *β*. It should be emphasized that (2) is only an illustrative example, whereas the overall concept outlined here is generic and applicable to other EM-driven tasks.

**Figure 1:**
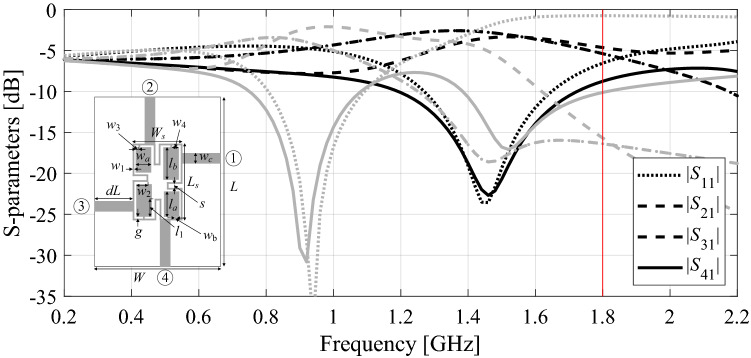
S-parameters of a compact branch-line coupler. Vertical line indicates the intended operating frequency of 1.8 GHz, which is reachable by a local optimizer if launched from the design marked black. Yet, it cannot be reached from the design marked grey due to the operating frequency overly distant from the intended one.

Figure 1 shows the example of a branch-line coupler, which is intended to work at 1.8 GHz. Local search starting from the design indicated using black lines will succeed, whereas optimization from the design shown using the grey lines will fail because of a significant discrepancy between the target and existent operating frequencies.

**Figure 2:**
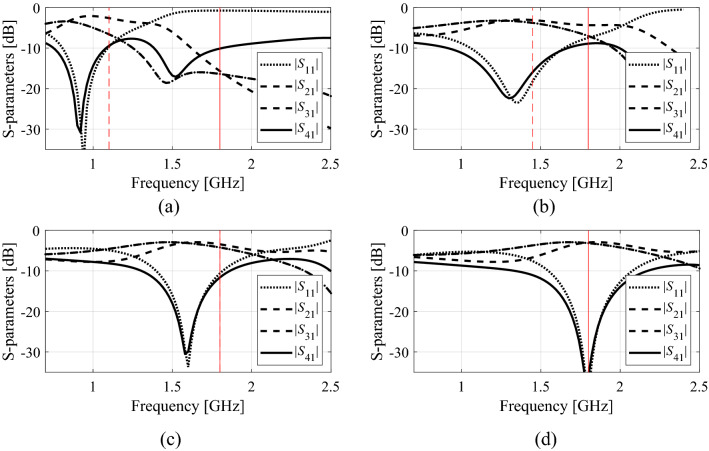
The concept of the design requirement adjustment on the example of a branch-line coupler of Fig. 1. The starting point (marked gray) and the intended operating frequency (marked using vertical line) are identical to that of Fig. 1: (**a**) target frequency shifted near the actual operating frequency of the initial design to make sure that the ongoing specifications (dashed line) may be reached from this very design, (**b**) intermediate step with the current design and specs shown, (**c**) ultimate optimization step: the intended operating frequency restored to its assumed value, (**d**) final design meeting the original requirements.

The automated adaptive specification management scheme^70^ addresses the above issue by relocating the targets throughout the optimization run so that they are reachable at each stage of the process. The amount of relocation depends on the detected discerned discrepancy between the existent and desired operating frequencies. A graphical illustration of the specification management procedure has been provided in Fig. 2.

### Automated adaptation of design requirements: prerequisites and implementation

The design specification management scheme operates based on the following prerequisites: (i) the necessary relocation of the target frequencies should be identified using the actual operating conditions at the current design, and (ii) the relocated goals should be reachable through local search.

In the following, ***J***(***x***) will represent the sensitivity matrix of the system outputs ***S***(***x***). Assuming that the optimization procedure is iterative and yields approximate designs ***x***^(*i*)^, *i* = 0, 1, …, to the optimum solution x^*^ of (1) (***x***^(0)^ denotes the starting point), we utilize the first-order linear expansion model *L*_*S*_^(*i*)^(***x***) of ***S***(***x***) at ***x***^(*i*)^

3 $$L_{S}^{(i)} ({\boldsymbol{x}}) = {\boldsymbol{S}}({\boldsymbol{x}}^{(i)} ) + {\boldsymbol{J}}({\boldsymbol{x}}^{(i)} ) \cdot ({\boldsymbol{x}} - {\boldsymbol{x}}^{(i)} )$$

Also, consider an auxiliary sub-problem

4 $${\boldsymbol{x}}^{tmp} = \arg \mathop {\min }\limits_{{||{\boldsymbol{x}} - {\boldsymbol{x}}^{(i)} || \le D}} U(L_{S}^{(i)} ({\boldsymbol{x}}),{\boldsymbol{F}})$$

where the search radius *D* is typically set to *D* = 1, whereas ***x***^*tmp*^ denotes the temporary design.

The automated alteration of design specs is based on the factors described in Table 1^70^.These guide the decision-making process and are used to define the conditions gathered in Table 2, satisfaction of which decides upon modification of the design goals with respect to their original values aggregated in the target vector ***F***. In particular, satisfaction of any of the conditions is considered an indication that the performance requirements are unlikely to be attained and should be relaxed accordingly.

**Table 1:** Adaptive performance specifications: decision factors.

Decision factor	Analytical formulation	Comments
Improvement factor	$F_{r} = \left| {U(L_{S}^{(i)} ({\boldsymbol{x}}^{tmp} ),{\boldsymbol{F}}) - U(L_{S}^{(i)} ({\boldsymbol{x}}^{(i)} ),{\boldsymbol{F}})} \right|$	Determines potential for design improvement starting from ***x***^(*i*)^
Distance between the actual and target operating frequencies	$D_{c} = ||{\boldsymbol{F}}_{c} - {\boldsymbol{F}}||$ where ***F***_*c*_ = [*f*_*c.*1_ … *f*_*c.N*_]^*T*^ (actual operating frequencies) ***F*** = [*f*_1_ … *f*_*N*_]^*T*^ (target frequencies)	Used as a safeguard to ensure that the updated specifications are sufficiently close to the current operating frequencies

**Table 2:** Design specification adjustment conditions^&^.

#	Condition^$^	Comment
1	*F*_*r*_ < *F*_*r*.min_	*F*_*r*_ is too small = > current design is not likely to be improved sufficiently when starting from ***x***^(*i*)^
2	*D*_*c*_ > *D*_*c*.max_	*D*_*c*_ is too large = > the operating frequencies at ***x***^(*i*)^ are too far away from the current targets

^&^Design specifications will be subject to modification if either of the conditions is satisfied.

^$^*F*_*r.*min_ and *D*_*c.*max_ are the user-defined acceptance thresholds.

The thresholds *F*_*r.*min_ (for improvement factor) and *D*_*c.*max_ (for distance between the actual and target operating frequencies) of Table 2 are generally problem dependent, and should be set up having in mind the typical (or expected) operating bandwidths of the circuit at hand. A simple procedure for adjusting these values has been described in Koziel et al.^70^.

Having defined the decision factors and adjustment conditions, we can now summarize the design specification management procedure. The target operating frequencies for the (*i* + 1)th iteration of the optimization algorithm will be denoted as ***F***_*current*_(*a*) = [*f*_*current.*1_(*a*) … *f*_*current.N*_(*a*)]^*T*^, where 0 ≤ *a* ≤ 1. The individual frequencies *f*_*current.k*_ are obtained as

5 $$f_{current.k} (a) = (1 - a)f_{c.k} + af_{k} \quad for\quad k = 1, \ldots ,N$$

where *f*_*c.k*_ are the existent operating frequencies at ***x***^(*i*)^ (we also have the vector of current operating frequencies ***F***_*c*_ = [*f*_*c.*1_ … *f*_*c.N*_]^*T*^). The value of the factor *a* is determined as the maximal value *a* ≤ 1 so that *F*_*r*_ ≥ *F*_*r*.min_ and *D*_*c*_ ≤ *D*_*c*.max_ at the design ***x***^*tmp*^ obtained through minimizing (cf. (4))

6 $${\boldsymbol{x}}^{tmp} = \arg \mathop {\min }\limits_{{||{\boldsymbol{x}} - {\boldsymbol{x}}^{(i)} || \le 1}} U(L_{S}^{(i)} ({\boldsymbol{x}}),{\boldsymbol{F}}_{current} (a))$$

In practice, *a* is found using an auxiliary numerical optimization process, in which it is gradually reduced (starting from *a* = 1) so that *F*_*r*_ ≥ *F*_*r*.min_ and *D*_*c*_ ≤ *D*_*c*.max_ for ***x***^*tmp*^ produced by (6). Satisfaction of both conditions means that the current targets are reachable from ***x***^(*i*)^. In the course of the optimization run, the adjusted specs will ultimately converge to the assumed values (which is equivalent to satisfying both *F*_*r*_ ≥ *F*_*r*.min_ and *D*_*c*_ ≤ *D*_*c*.max_ for *a* = 1), assuming that that initial specs are attainable. Otherwise, the algorithm will terminate when getting as close to the targets as achievable.

The described decision-making procedure is executed before each iteration of the search process. Consequently, the design targets are continuously adjusted to account for the current discrepancies between the actual and desired operating parameters. An important observation is that the modification process incurs no extra computational costs (in terms of additional EM evaluations), because it is based on the sensitivity data already evaluated during routine working of the optimization procedure.

## Variable-fidelity models for optimization cost reduction

In this work, the primary tool incorporated to enhance computational efficacy of the optimization process is the incorporation of variable-fidelity EM simulation models. This section explicates the introduced knowledge-based model fidelity management procedure, which is based on two factors: (1) the detected discrepancy between the current and original design targets, and (2) the convergence indicators of the algorithm.

### Variable-fidelity EM simulations

Computational models of microwave devices can be implemented using full-wave EM analysis^71^, or circuit theory tools (equivalent networks^72^, analytical descriptions^73^). In this work, it is assumed that the primary (high-fidelity) representation of the circuit of interest is in the form of (high-fidelity) EM simulation, whereas lower-fidelity models are obtained through EM analysis executed at lower discretization density of the structure. This is a versatile and easy to control way, which also ensures a sufficiently good correlation between the models of different resolutions. Other simplification factors (e.g., neglecting losses, reducing computational domain^74^) will not be considered here. Utilization of multi-fidelity models can be beneficial for computational efficiency of the CAD procedures, e.g., space mapping^75^, response correction^45,46^, co-kriging^76^. Typically, two levels of fidelity are employed (coarse/low-fidelity, fine/high-fidelity)^77^), which raises some practical issues related to appropriate model selection and setup^78^.

**Figure 3:**
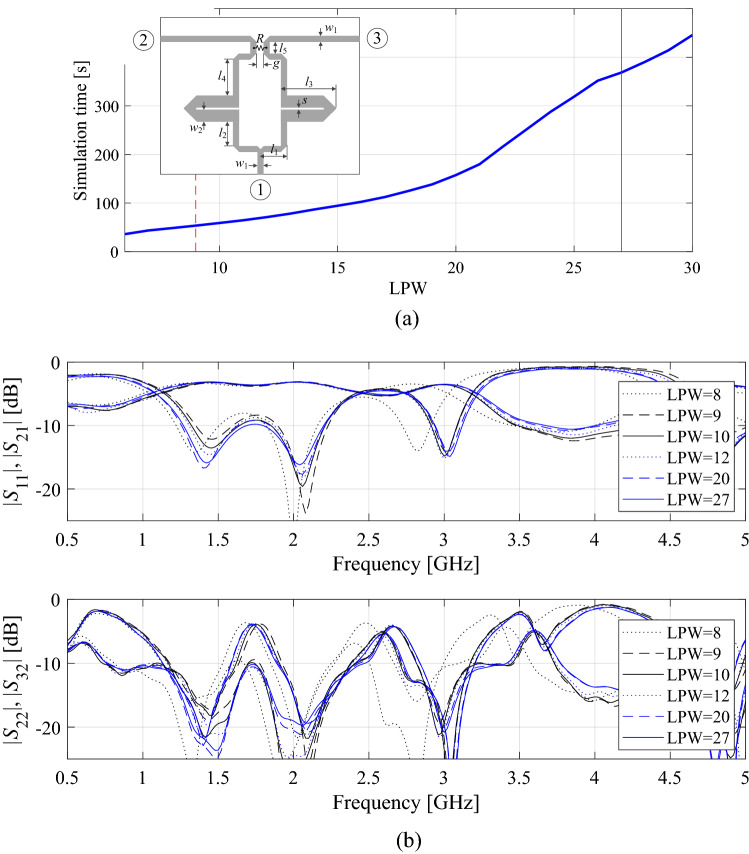
Dual-band power divider with multi-fidelity EM simulations: (**a**) average simulation time vs model resolution (controlled by LPW, i.e., lines-per-wavelength parameter), (**b**) certain S-parameters for the chosen LPW values. The vertical lines denote resolutions of the fine (high-fidelity) model (—), and the coarse (low-fidelity) model (---).

Model fidelity can be modified using the parameters controlling the meshing algorithms, e.g., lines-per-wavelength (LPW) of CST Microwave Studio. Figure 3 provides an example of a dual-band power divider and the relationship between LPW and the average simulation times of the structure. The minimum model fidelity (here, denoted as *L*_min_) should be selected to ensure that the corresponding circuit responses are still representative, i.e., not excessively distorted with respect to the maximum fidelity (here, denoted as *L*_max_). The latter, corresponding to the high-fidelity model, should render the circuit response of the accuracy considered sufficient for practical purposes. Observe that in our work, the model accuracy is understood as the accuracy of the EM simulation model implemented is CST Microwave Studio, and it depends on the mesh density. In general, the higher the density, the better the model accuracy.

The computational model fidelity *L* is selected from the admissible range *L*_min_ ≤* L* ≤ *L*_max_. At the initial phase of the optimization procedure, *L* is set to *L*_min_ so as to accelerate the optimization process. Towards the end of the run, *L* gradually converges to *L*_max_ to ensure reliability. The resolution at any given iteration of the optimization algorithm is governed by the discrepancy between the original and current values of the target operating frequencies (cf. “Knowledge-based model fidelity adjustment based on performance specifications”section), and by the algorithm convergence status (cf. “Model fidelity adjustment based on convergence status”section).

### Knowledge-based model fidelity adjustment based on performance specifications

Here, we propose to adjust the model fidelity at the initial phase of the optimization run based on the distance *D*_*cr*_ =||***F***_*cr*_ − ***F***|| between the target operating vector ***F***_*cr*_ at the current iteration point and the target ***F***. It should be noted that *D*_*cr*_ is similar to *D*_*c*_ (cf. Table 1), but evaluated using the modified targets instead of the current operating frequencies ***F***_*c*_. Further, we denote *D*_*cr*_^(0)^ as the value of *D*_*cr*_ after the initial adjustment of the targets, which will be the point of reference for subsequent fidelity modifications. At this point, the fidelity parameter *L* is set to *L*_min_ (i.e., the optimization process is initiated with the lowest-fidelity model). The model fidelity *L*^(*i*)^ at the *i*th iteration is set according using the formula

7 $$L^{(i)} = L_{\min } + \alpha \left( {L_{\max } - L_{\min } } \right)\left[ {1 - \frac{{D_{cr}^{(i)} }}{{D_{cr}^{(0)} }}} \right]$$

where ***F***_*cr*_ denotes the target operating vector at the current iteration point, $D_{cr}^{(i)} = ||{\boldsymbol{F}}_{cr}^{(i)} - {\boldsymbol{F}}||$; here, and ***F***_*cr*_^(*i*)^ stands for the target operating frequencies modified for iteration *i*. The scalar coefficient *α* ∈ [0, 1] controls the maximum model fidelity used until ***F***_*cr*_^(*i*)^ reaches ***F*** (for the experiments of “Verification case studies” section, we set *α* = 0.5).

### Model fidelity adjustment based on convergence status

The increase of the model fidelity is continued after reducing *D*_*cr*_^(*i*)^ to zero, i.e., after ***F***_*cr*_^(*i*)^ becomes equal to ***F***. This second stage is governed by the procedure discussed in^79^. It is also assumed that the optimization process is concluded if one of the two conditions is met: (i) ||***x***^(*i*+1)^ − ***x***^(*i*)^||< *ε*_*x*_ (convergence in argument), or |*U*(***x***^(*i*+1)^) − *U*(***x***^(*i*)^)|< *ε*_*U*_ (convergence in the merit function value). Therein, *ε*_*x*_ and *ε*_*U*_ are the termination thresholds, set to *ε*_*x*_ = 10^−3^ and *ε*_*U*_ = 10^−2^, in the numerical experiments of “Verification case studies” section. Let us also consider the convergence factor^79^

8 $$Q^{(i)} (\varepsilon_{x} ,\varepsilon_{U} ) = \max \left\{ {\varepsilon_{x} /||{\boldsymbol{x}}^{(i + 1)} - {\boldsymbol{x}}^{(i)} ||,\varepsilon_{U} /|U_{P} ({\boldsymbol{x}}^{(i + 1)} ) - U_{P} ({\boldsymbol{x}}^{(i)} )|} \right\}$$

It is employed to decide upon the model fidelity level *L*^(*i*+1)^ for the (*i* + 1)th algorithm iteration. We have

9 $$L^{(i + 1)} = \left\{ \begin{gathered} L_{cr} \;\;{\text{if}}\;\;Q^{(i)} (\varepsilon_{x} ,\varepsilon_{U} ) \le M \hfill \\ \max \left\{ {L^{(i)} ,L_{cr} + \left( {L_{\max } - L_{cr} } \right)\left[ {1 - \frac{{\log (Q^{(i)} (\varepsilon_{x} ,\varepsilon_{U} )}}{\log M}} \right]} \right\} \hfill \\ \end{gathered} \right.$$

where *L*_*cr*_ is the model fidelity when the actual frequency ***F***_*cr*_^(*i*)^ first reached the target ***F***. The parameter *M* determined the convergence level for initiating fidelity adjustment (set *M* = 10^2^ε_*x*_, as recommended in^79^). Furthermore, the fidelity is obligatorily set to *L*_max_ near the convergence if the highest *L*^(*i*)^ was below *L*_max_. In such case, the search region size (cf. “Trust-region-embedded gradient-search” section) is additionally extended by a multiplier *M*_*d*_ (we use, *M*_*d*_ = 10), and the search continues with *L*^(*i*+1)^ = *L*_max_^79^.

The final acceleration mechanism is to evaluate the Jacobian matrices of the circuit at hand through finite differentiation executed at iteration *i* at lower level of fidelity *L*_*FD*_ (model fidelity for carrying out finite differentiation), rather than *L*^(*i*)^. Here, *L*_*FD*_ = max{*L*_min_, *λL*^(*i*)^}, with *λ* = 2/3 (cf.^79^). It has been observed that this typically results in reducing the overall computational cost (due to good correlation of sensitivities for models of different resolutions), even though the optimization may require a slightly larger number of iterations.

## Complete optimization framework

Here, we put together the algorithmic components discussed in “Adaptive design requirements for reliability improvement” and “Variable-fidelity models for optimization cost reduction” sections, and summarize the operation of the entire procedure proposed in this work. The core optimization procedure is a gradient-based routine with numerical derivatives, which will be recalled in “Trust-region-embedded gradient-search” section. “Optimization algorithm” section provides the pseudocode of our algorithm, along with the flow diagram thereof.

### Trust-region-embedded gradient-search

The algorithmic components oriented towards improving the reliability (“Adaptive design requirements for reliability improvement” section) and computational efficiency of the search process (“Variable-fidelity models for optimization cost reduction” section) can be incorporated into any iterative optimization procedure. In this work, the core routine is the trust-region (TR) gradient search^80^. The design task is the minimization problem (1). The TR algorithm works iteratively and yields a series of approximations ***x***^(*i*)^, *i* = 0, 1, …, to the optimum design x^*^ as

10 $${\boldsymbol{x}}^{(i + 1)} = \arg \mathop {\min }\limits_{{||{\boldsymbol{x}} - {\boldsymbol{x}}^{(i)} || \le d^{(i)} }} U(L_{S}^{(i)} ({\boldsymbol{x}}),{\boldsymbol{F}}_{cr}^{(i)} )$$

In (10), *L*^(*i*)^ is a linear expansion model (3) established at the current design ***x***^(*i*)^. Recall that ***F***_*cr*_^(*i*)^ is the current vector of assumed operating frequencies. Throughout the optimization run, the update of the TR search radius *d*^(*i*)^ is performed iteratively by taking into account the gain ratio *r* = [*U*(***S***(***x***^(*i*+1)^),***F***_*cr*_^(*i*)^) − *U*(***S***(***x***^(*i*)^),***F***_*cr*_^(*i*)^)]/[*U*(*L*_*S*_^(*i*)^(***x***^(*i*+1)^),***F***_*cr*_^(*i*)^) − *U*(*L*_*S*_^(*i*)^(***x***^(*i*)^),***F***_*cr*_^(*i*)^)], which quantifies the actual improvement of the objective function (based on EM analysis) versus the estimated improvement (based on the linear model prediction). In case of improvement (*r* > 0), the design ***x***^(*i*+1)^ is accepted. Also, if *r* > 0.75, *d*^(*i*+1)^ is increased to 2*d*^(*i*)^; if *r* < 0.25, *d*^(*i*+1)^ is reduced to *d*^(*i*)^/3. Rejection of the design (*r* < 0) results in repeating the iteration with a reduced TR size.

### Optimization algorithm

**Figure 4:**
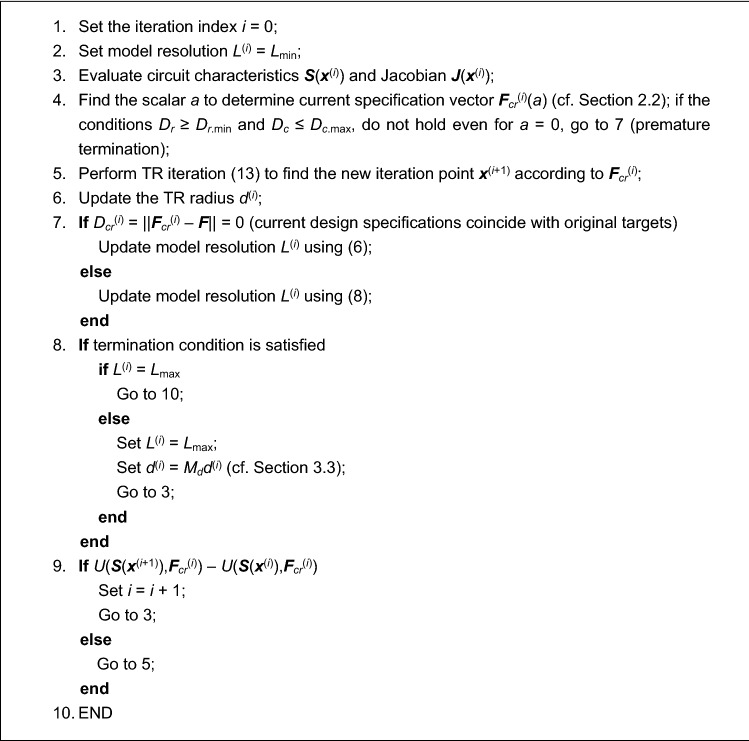
Pseudocode of the proposed optimization algorithm with design requirement management and variable-fidelity EM models.

**Figure 5:**
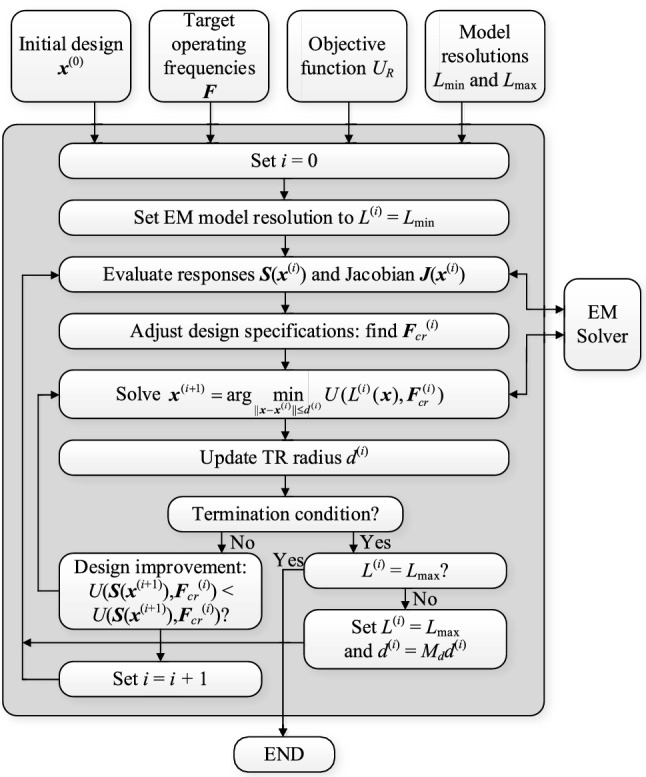
Flow diagram of the introduced optimization algorithm with design requirement adjustment and variable-fidelity EM models.

The kernel of the knowledge-based optimization procedure introduced in this paper is the TR algorithm briefly discussed in “Trust-region-embedded gradient-search” section. The automated design requirement management strategy of “Adaptive design requirements for reliability improvement” section, and the variable-fidelity model adjustment scheme of “Variable-fidelity models for optimization cost reduction” section, are simultaneously incorporated therein. In particular, both the design goals and the model fidelity are adjusted before each iteration of the TR routine. The goals are modified based on the decision factors of Table 1 and conditions of Table 2, whereas the model fidelity is altered using the coefficient *D*_*cr*_ (cf. “Knowledge-based model fidelity adjustment based on performance specifications” section), and the convergence indicator *Q*^(*i*)^ (cf. “Model fidelity adjustment based on convergence status” section). Figure 4 shows the pseudocode of the entire procedure, whereas Fig. 5 provides its flow diagram. The designer needs to supply the following information:

- Initial design ***x***^(0)^,
- Analytical formula for the objective function *U*,
- Target vector ***F***,
- The range of EM model fidelities *L*_min_ and *L*_max_.

Also, the termination condition discussed in “Model fidelity adjustment based on convergence status” section (argument and objective function convergence) needs to be complemented by an additional condition specific to the trust region frameworks, i.e., *d*^(*i*)^ < *ε*_*x*_ (reduction of the TR size).

## Verification case studies

The algorithm introduced in “Adaptive design requirements for reliability improvement” through “Complete optimization framework” sections is verified here with the use of three examples of microstrip circuits: two branch-line couplers (a single- and dual-band one), and a dual-band power divider. All circuits are optimized from inferior-quality initial designs, i.e., such whose operating frequencies are away from the design targets. This setup allows us to demonstrate the relevance of the reliability improvements achieved through the adaptive performance requirement approach. At the same time, we investigate computational savings that can be obtained using the variable-fidelity mechanisms incorporated into our procedure. All the simulations were performed on Intel Xeon 2.1 GHz dual-core CPU with 128 GB RAM.

### Circuit I: compact branch-line coupler (BLC)

**Figure 6:**
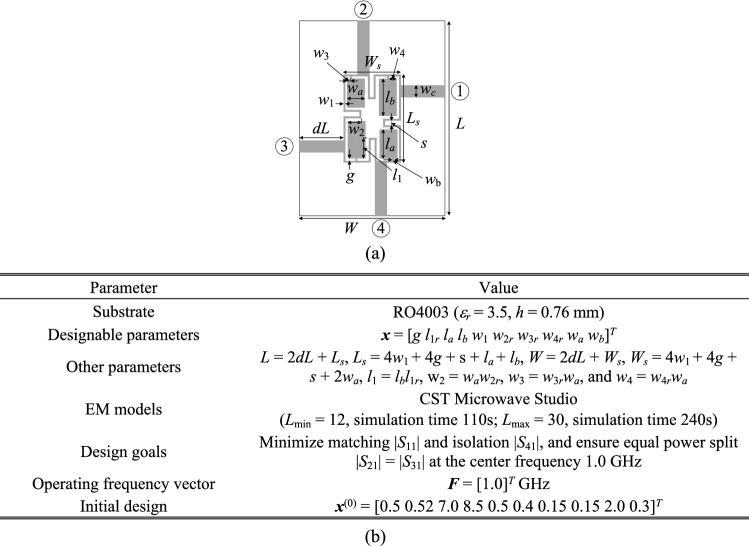
Compact branch-line coupler (Circuit I): (**a**) geometry^81^; (**b**) main parameters and design objectives.

**Figure 7:**
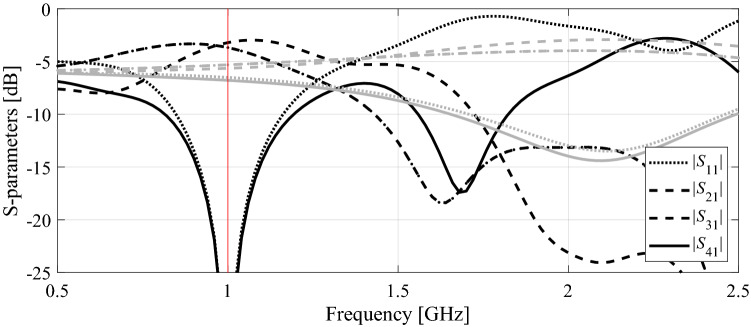
Compact branch-line coupler: circuit responses at the initial design (grey lines), and the optimal design rendered by the introduced framework with design specification adaptation and variable-fidelity models (black lines). Vertical line marks target operating frequency.

**Figure 8:**
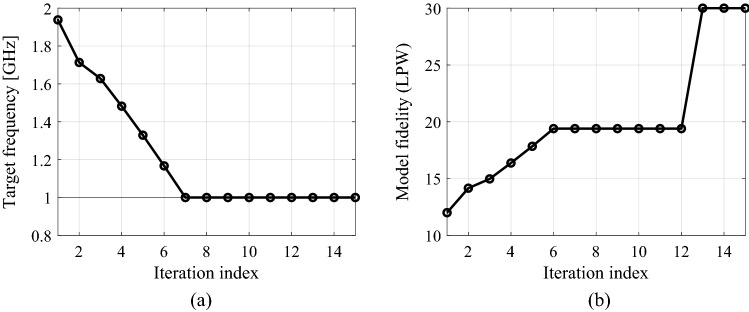
Compact branch-line coupler: (**a**) history of the target operating frequency (horizontal line marks the initial target); (**b**) evolution of the model resolution.

The first example is a compact branch-line coupler shown in Fig. 6a. Figure 6b provides the relevant data, including designable parameters, computational models, initial design, and performance specifications. The circuit is to be optimized to minimize its matching and port isolation, as well as to provide equal power split at the center frequency of 1.0 GHz. The results obtained using the proposed algorithm, standard gradient-based optimization (cf. "Trust-region-embedded gradient-search" section), as well as adaptive design requirements technique^70^, have been gathered in Table 3. The *S*-parameters of the circuit at the initial design as well as design obtained using the presented approach can be found in Fig. 7. The optimized parameter values are x^*^ = [0.99 0.65 8.59 13.2 1.00 0.94 0.85 0.62 4.02 0.24]^*T*^ mm. It can be noted (cf. Table 3) that the designs obtained using the algorithm discussed in this work and the method of Koziel et al.^70^ are of similar quality. Moreover, the computational speedup achieved through the incorporation of variable-fidelity EM simulations is significant: the total cost of the parameter tuning process corresponds to only 97 high-fidelity circuit analyses (51 percent savings over^70^). As indicated in Table 3, conventional gradient-based search failed to identify a satisfactory design. The evolution of the design targets and model fidelity has been illustrated in Fig. 8. Note that the major part of the optimization process has been carried out using lower-fidelity models, the high-fidelity simulations are only applied at the latest stages of the algorithm, which translated into the aforementioned speedup.

**Table 3:** Optimization results for Circuit I.

Algorithm	Operating frequency at the optimized design	Optimization cost^#^	Cost savings over algorithm of Koziel et al.^70^*
Conventional TR procedure (cf. "Trust-region-embedded gradient-search" section)	N/A^&^	N/A^&^	N/A^&^
Adaptive performance specifications^70^	1.0 GHz	198	–
Variable-fidelity adaptive performance specifications (this work)	1.0 GHz	97	51.0%

^$^Objective function computed as in (2).

^#^Cost expresses in equivalent number of high-fidelity EM simulations.

^&^The algorithm failed to identify a satisfactory design, in particular, align the circuit operating frequency with the target.

*Relative computational savings in percent w.r.t. the algorithm of Koziel et al.^70^.

### Circuit II: dual-band branch-line coupler

**Figure 9:**
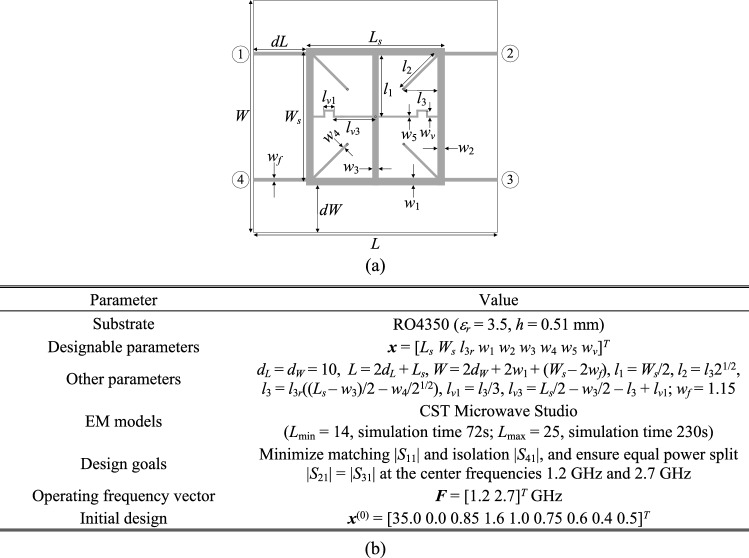
Dual-band branch-line coupler (Circuit II): (**a**) geometry^82^; (**b**) main parameters and design objectives.

**Figure 10:**
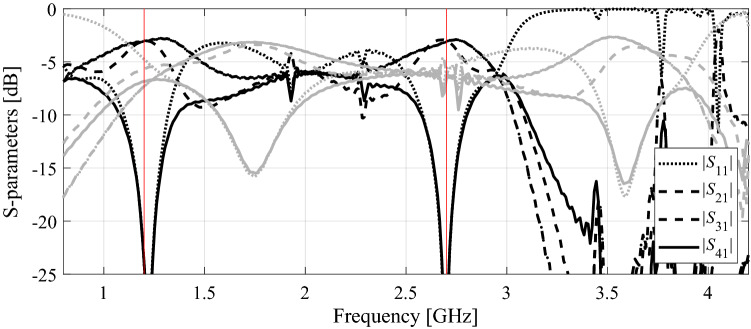
Dual-band branch-line coupler: circuit responses at the initial design (grey lines), and the optimal design rendered by the introduced framework with design specification adaptation and variable-fidelity models (black lines). Vertical lines mark target operating frequencies.

**Figure 11:**
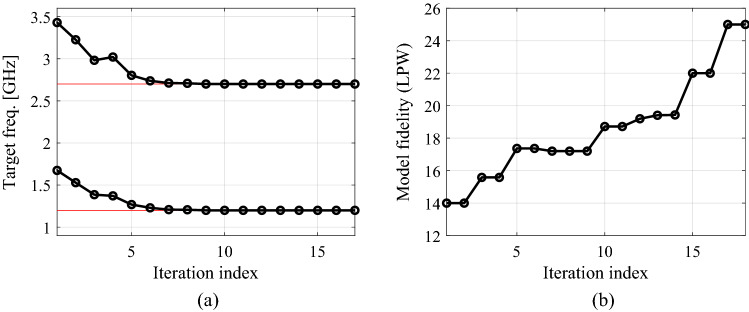
Dual-band branch-line coupler: (**a**) history of the target operating frequency (horizontal lines mark the initial targets); (**b**) evolution of the model resolution.

As the second verification case, consider a dual-band branch-line coupler of Fig. 9a. The important parameters of the circuit have been listed in Fig. 9b. In this case, the design objective is to minimize the matching |*S*_11_| and isolation |*S*_41_|, and to achieve equal power split at the operating frequencies of 1.2 GHz and 2.7 GHz. Table 4 gathers the optimization results for the introduced and the benchmark methods. Figure 10 shows the coupler *S*-parameters at the initial and the final design, x^*^ = [42.0 10.0 0.85 2.56 1.50 1.33 0.60 0.44 2.01]^*T*^ mm, found by the algorithm of "Verification case studies" section. Similarly as for the first example, the utilization of variable-fidelity simulations leads to considerable computational savings of 61 percent over the adaptive design specification method of Koziel et al.^70^. The cost reduction is achieved without compromising the design quality as indicated in Table 4. In absolute terms, optimization cost corresponds to 94 EM analyses of the coupler using highest resolution. Figure 11 illustrates the evolution of design goals and model fidelity.

**Table 4:** Optimization results for Circuit II.

Algorithm	Operating frequency at the optimized design	Optimization cost^#^	Cost savings over algorithm of Koziel et al.^70^*
Conventional TR procedure (cf. "Trust-region-embedded gradient-search" section)	N/A^&^	N/A^&^	N/A^&^
Adaptive performance specifications^70^	[1.2 2.7] GHz	243	–
Variable-fidelity adaptive performance specifications (this work)	[1.2 2.7] GHz	94	61.3%

^$^Objective function computed as in (2).

^#^Cost expresses in equivalent number of high-fidelity EM simulations.

^&^The algorithm failed to identify a satisfactory design, in particular, align the circuit operating frequency with the target.

*Relative computational savings in percent w.r.t. the algorithm of^70^.

### Circuit III: dual-band power divider

**Figure 12:**
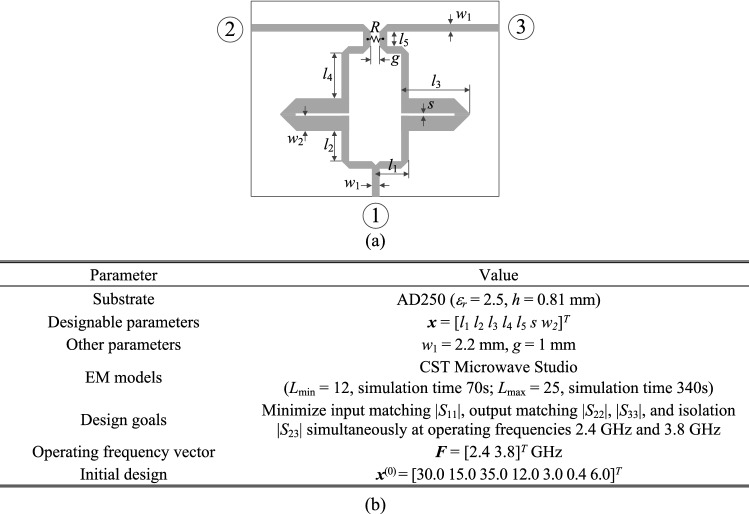
Dual-band power divider (Circuit III): (**a**) geometry^83^; (**b**) main parameters and design objectives.

**Figure 13:**
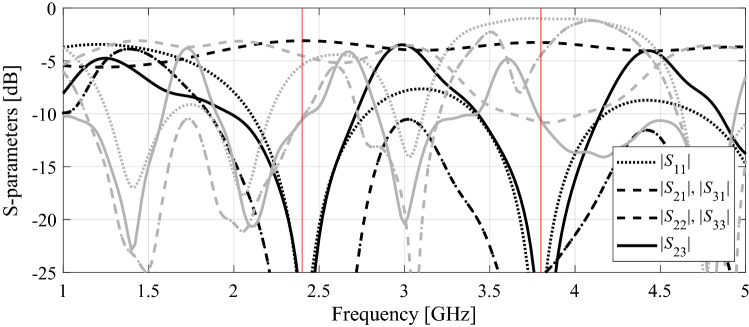
Dual-band power divider: circuit responses at the initial design (grey lines), and the optimal design rendered by the introduced framework with design specification adaptation and variable-fidelity models (black lines). Vertical lines mark target operating frequencies.

**Figure 14:**
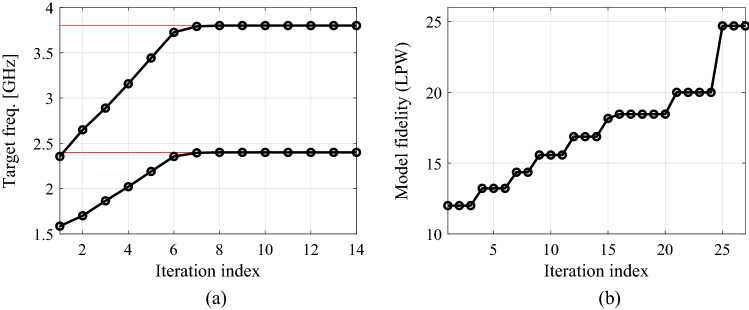
Dual-band power divider: (**a**) history of the target operating frequency (horizontal lines mark the initial targets); (**b**) evolution of the model resolution.

The final verification case is a dual-band power divider shown in Fig. 12a. The essential circuit parameters have been provided in Fig. 12b. The aim is to minimize the input and output matching (|*S*_11_|, |*S*_22_|, |*S*_33_|) and port isolation |*S*_23_| simultaneously at the operating frequencies 2.4 GHz and 3.8 GHz, as well as to obtain equal power division ratio. The latter is implied by the circuit symmetry, therefore, does not have to be explicitly handled in the optimization process. The numerical results are provided in Table 5. The algorithm performance is in accordance with that of the previous examples. On the one hand, we observed considerable computational savings of 54 percent over the single-fidelity procedure of Koziel et al.^70^. On the other hand, the quality of design produced by the presented method is similar to the benchmark. It should also be noted that the conventional gradient search fails due to severe misalignment between operating frequencies of the coupler at the initial design and the assumed ones. The optimized parameter vector is x^*^ = [26.3 5.09 20.6 5.12 1.0 0.60 4.34]^*T*^. The remaining results can be found in Fig. 13 (circuit responses at the initial and optimal designs), and Fig. 14 (evolution of the design specifications and model fidelity).

**Table 5:** Optimization results for Circuit III.

Algorithm	Operating frequency at the optimized design	Optimization cost^#^	Cost savings over algorithm of Koziel et al.^70^*
Conventional TR procedure (cf. "Trust-region-embedded gradient-search" section)	N/A^&^	N/A^&^	N/A^&^
Adaptive performance specifications^70^	[2.4 3.8] GHz	274	–
Variable-fidelity adaptive performance specifications (this work)	[2.4 3.8] GHz	125	54.4%

^$^Objective function computed as in (2).

^#^Cost expresses in equivalent number of high-fidelity EM simulations.

^&^The algorithm failed to identify a satisfactory design, in particular, align the circuit operating frequency with the target.

*Relative computational savings in percent w.r.t. the algorithm of Koziel et al.^70^.

The introduced approach is an accelerated version of the algorithm proposed in Koziel et al.^70^. In contrast to^70^, where only single-fidelity EM model of the component under design is employed, here, we utilize EM models of various fidelities belonging to the continuous range of admissible resolutions. This is a source of the computational benefits of our procedure over that proposed in Koziel et al.^70^. The reliability of our procedure is excellent: it has been capable of yielding the designs fulfilling the required design specifications in all the considered cases, even though the starting points have been to a large extent misaligned with the targets. At the same time, the speedup over the single-fidelity framework^70^ is around fifty-five percent on average (from fifty to sixty percent across the benchmark set).

## Conclusion

In this work, we proposed a new technique for computationally-efficient and improved-reliability parameter tuning of microwave passive components. The presented approach combines two distinct algorithmic tools, the automated design requirement management scheme, and the knowledge-based adaptively-adjusted EM simulation fidelity mechanism. The former allows for a considerable improvement of the optimization process reliability. In particular, it enables successful local tuning even under challenging conditions (e.g., poor starting point). The latter results in a significant computational speedup with respect to the standard, single-fidelity optimization. Both mechanisms are implemented to work simultaneously. More specifically, the decision-making procedure governing model fidelity setup for a given iteration of the optimization algorithm depends on the current discrepancy between the observed and target operating parameters of the circuit at hand, as well as the convergence status of the search process. The proposed framework is intended to work with full-wave simulation models (e.g., finite-difference time-domain (FDTD), or finite element method (FEM)), but also dedicated solvers that permit a control over discretization density of the structure under simulation. The prerequisite is that the utilized computational models should be evaluated using the same simulation engine, and differ solely by mesh density to ensure satisfactory correlation between the models of different resolutions. Whereas this level of correlation is not possible to achieve with circuit-theory models (or equivalent circuits, or else analytical models). The methodology proposed in this work has been validated using three microstrip components, including two couplers and a power divider. In all cases, it demonstrated superior performance, both in terms of successful allocation of the operating frequencies of the considered circuits despite of poor initial designs, and computational efficiency. The average CPU savings over the recent technique involving adaptively adjusted design specifications are as high as 55 percent. The speedup has been shown not to be detrimental to the design quality. The optimization strategy introduced in this paper has a potential to replace or complement traditional methods, especially in situations where local optimization is likely to fail due to the lack of good starting points or the necessity of re-designing the circuit over broad frequency ranges, whereas the involvement of global search routines is questionable because of the incurred computational expenses.

## Data Availability

The datasets generated during and/or analysed during the current study are available from the corresponding author on reasonable request. Contact person: anna.dabrowska@pg.edu.pl.
